# Bacteriologic Profile Along With Antimicrobial Susceptibility Pattern of Pediatric Pyoderma in Eastern India

**DOI:** 10.7759/cureus.25716

**Published:** 2022-06-07

**Authors:** Somosree Ghosh, Mallika Sengupta, Soma Sarkar, Sampurna Biswas Pramanik, Manideepa Sengupta, Debabrata Bandyopadhyay

**Affiliations:** 1 Microbiology, KPC Medical College, Kolkata, IND; 2 Microbiology, All India Institute of Medical Sciences, Kalyani, Kalyani, IND; 3 Microbiology, Nil Ratan Sircar Medical College, Kolkata, IND; 4 Microbiology, Medical College Kolkata, Kolkata, IND; 5 Dermatology, Medical College Kolkata, Kolkata, IND

**Keywords:** methicillin-resistant staphylococcus aureus (mrsa), antibiogram, pyoderma, coagulase, scabies

## Abstract

Background

Pyogenic skin infection (pyoderma) is a bacterial infection of the skin and its appendages. Primary pyoderma is caused by the direct invasion of healthy skin, whereas secondary pyoderma originates in diseased skin as superimposed conditions, such as scabies, pediculosis, wounds, insect bites, and eczema. This study aimed to identify the clinical patterns and risk factors of pyoderma in a pediatric population and to isolate various causative bacteria and determine their susceptibility patterns.

Methodology

A prospective study was performed at the Medical College and Hospital, Kolkata, India, for one year (from August 2016 to July 2017), which included all children younger than 12 years with pyoderma attending the outpatient dermatology department (as the study was conducted among the pediatric population, only children below 12 years of age were included). Sterile cotton swabs were used to aseptically collect exudates or pus from lesions and anterior nares, which were used for culture, identification, and antibiotic susceptibility testing of the causative organisms.

Results

During the study period, a total of 182 patients were included, 121 (66.48%) of whom had primary pyoderma and 61 (33.52%) of whom had secondary pyoderma. Of the 182 patients, 161 showed bacterial growth on culture: 126 (78.26%) were *Staphylococcus aureus*, 18 (11.18%) were coagulase-negative staphylococci, 16 (9.94%) were *Streptococcus pyogenes*, and 1 (0.62%) was *Pseudomonas aeruginosa*. All staphylococci were susceptible to vancomycin and linezolid.

Conclusions

The most common cause of pyoderma in the pediatric age group is *S. aureus*, although the prevalence of methicillin-resistant *S. aureus* was low in this hospital. Proper identification and antibiogram are required for managing these cases.

## Introduction

Pyoderma is defined as a purulent infection of the skin and constitutes one of the most common clinical conditions encountered by dermatologists. Pyoderma is classified as primary or secondary, with the former accounting for infection of healthy skin and the latter occurring from pre-existing skin disease [1]. Primary pyoderma includes impetigo, folliculitis, furunculosis, carbuncle, ecthyma, and sycosis, whereas secondary pyoderma includes infected scabies, infected eczema, infected wounds, and infected trophic ulcers [2]. Various factors have been implicated in the formation of pyoderma, such as overcrowding, poverty, malnutrition, poor hygiene, illiteracy, customs, low immunity, lifestyle habits, and various traumas, such as insect bites and thorn pricks [3]. Pyoderma is a common infection in the pediatric age group, and its most common causative agents are *Staphylococcus* and *Streptococcus* [4].

The spectrum of causative pyoderma agents and their antibiotic susceptibility patterns are constantly changing. Increasing antibiotic resistance hinders the effective management of such cases. More potent drugs should not be used for susceptible bacteria because they can lead to the spread of antibiotic resistance. For the successful treatment of pyoderma, detailed knowledge of its clinical patterns, its causative bacteria, and its contemporary antibiotic susceptibility pattern is needed.

This study aimed to identify the clinical pattern and risk factors of pyoderma in the pediatric population and to isolate, identify, and determine the antibiotic susceptibility pattern of the various causative bacteria in primary and secondary pyoderma. This study also determined the nasal carriage of *Staphylococcus*.

## Materials and methods

A prospective, cross-sectional study was performed at the Medical College and Hospital, Kolkata, a tertiary care center in Eastern India, for a period of one year (August 2016 to July 2017), after obtaining ethical clearance from the Institutional Ethics Committee of Medical College Kolkata (MC/KOL/IEC/NON-SPON/104/10-2015 dated 13-10-2015). All children 12 years of age or younger who were treatment-naïve and presented with pyogenic skin infection at the outpatient dermatology department were included in the study. Patients who had previously taken antibiotics for the disease concerned, those who were older than 12 years of age, or who had other infections (such as fungal infections) were excluded from this study.

After obtaining informed consent from the patients’ parents, a detailed history regarding the patients’ age, sex, living conditions, lesion morphology, illness duration, disease onset and progression, ingestion of any drugs, and associated metabolic disorders was recorded, and a thorough clinical examination was performed. Sterile cotton swabs were used to aseptically collect exudates or pus from the lesions. Two samples were taken from the patients’ lesions and two from their anterior nares. In the case of intact pustular lesions, the pustule was ruptured with a sterile needle and material was collected on two sterile swabs. One swab from each set (pus and anterior nares) was used for Gram staining of the smear, and the other was immediately inoculated on blood agar, MacConkey’s agar, and brain heart infusion broth. The inoculated media were incubated at 37°C and kept for 48 hours, with readings taken after 24 and 48 hours as per the standard guidelines.

Organism growth was identified by standard microbiological techniques, including Gram staining, relevant biochemical reactions, and the VITEK 2 Compact instrument (bioMerieux Inc., France). Antibiotic susceptibility testing was performed on Mueller-Hinton agar plates using the Kirby-Bauer disc diffusion method and was interpreted according to the Clinical and Laboratory Standards Institute guidelines (2016 version) [4]. The *Staphylococcus* isolates were tested with cefoxitin (30 µg), ciprofloxacin (5 µg), levofloxacin (5 µg), erythromycin (15 µg), clindamycin (2 µg), trimethoprim-sulfamethoxazole (1.25/23.75 µg), amikacin (30 µg), gentamicin (10 µg), doxycycline (30 µg), and linezolid (30 µg) using the disc diffusion method and with vancomycin and teicoplanin using the E-strip method. To determine inducible clindamycin resistance, a D-test was performed with erythromycin and clindamycin discs. All data were entered into an Excel spreadsheet (Microsoft, Seattle, WA, USA) and analyzed.

## Results

During the one-year study period (from August 2016 to July 2017), a total of 182 patients were included. The children were aged from two days to 12 years. There were 13 (7.15%) children younger than one year, 68 (37.36%) were one to four years of age, 52 (28.57%) were five to eight years of age, and 49 (26.92%) were nine to twelve years of age (mean age, 5.62 years). Of the 182 patients, 97 (53.29%) were male, 85 (46.71%) were female, 119 (65.38%) lived in an urban area, and 63 (34.62%) lived in a rural area.

A total of 121 (66.48%) patients had primary pyoderma, and 61 (33.52%) had secondary pyoderma. Among the primary pyoderma cases, impetigo contagiosa (34.07%) was the most common type, followed by folliculitis (20.33%), including both superficial and deep folliculitis, whereas among the secondary pyoderma cases, scabies with secondary infection (14.84%) was more common than eczema with secondary infection (11.54%). Pain, fever, and discharge were the most common clinical features observed in 118 (64.83%) cases, followed by itching and fever in 18 (9.89%) cases. The lesion distribution was more common in the head and neck region in 61 (33.52%) cases, followed by the lower limbs in 36 (19.78%) cases. Pustules were the most common type of lesion observed in 112 (61.54%) cases, followed by crusting and erosion in 30 (16.48%) cases (Table 1).

**Table 1:** The clinical presentation of pyoderma.

Clinical presentation	Number of patients (%)
Lesion site (n = 182)	
Head and neck	61 (33.52%)
Upper limbs	23 (12.64%)
Lower limbs	36 (19.78%)
Trunk	27 (14.83%)
Pelvic area, including genitalia	1 (0.55%)
Multiple	34 (18.68%)
Lesion type (n = 182)	
Pustule	112 (61.54%)
Nodule	8 (4.39%)
Crusting and erosion	30 (16.48%)
Edema	9 (4.95%)
Abscess	15 (8.25%)
Ulcer	8 (4.39%)
Associated symptoms (n = 182)	
Pain	6 (3.29%)
Pain and swelling	14 (7.69%)
Pain and fever	17 (9.35%)
Pain, fever, and discharge	118 (64.83%)
Itching	9 (4.95%)
Itching and fever	18 (9.89%)

**Figure 1:**
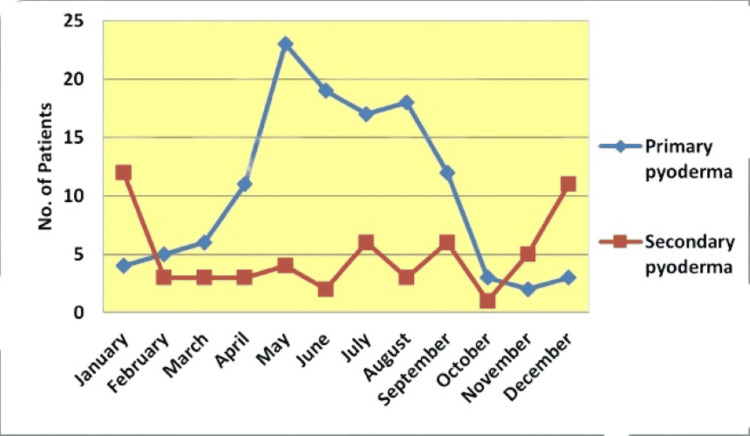
Line graph of the seasonal variations in clinically suspected primary and secondary pediatric pyoderma.

Primary pyoderma cases were more frequent in the hot and humid months of May, June, July, and August, whereas secondary pyoderma occurred more frequently in the winter months of December and January (Figure 1).

**Figure 2:**
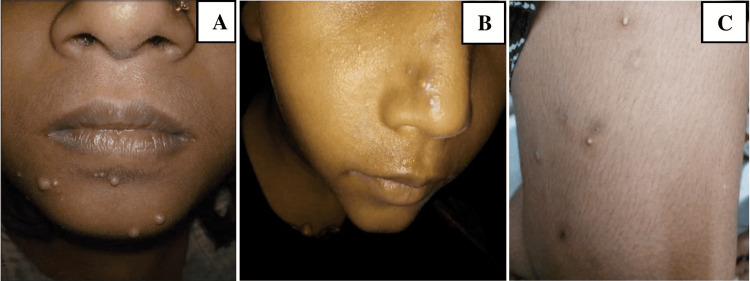
(A) Impetigo contagiosa; (B) impetigo contagiosa (nodular lesion); (C) impetigo contagiosa (pustular lesion).

Of the 182 patients, 161 (88.46%) showed bacterial growth in culture whereas 21 (11.54%) had no growth after a 48-hour incubation period. Of these 161 cases, 126 (78.26%) were *S. aureus*, 18 (11.18%) were coagulase-negative staphylococci, 16 (9.94%) were *S. pyogenes*, and 1 (0.62%) was *P. aeruginosa*. The most common clinical condition was impetigo contagiosa (Table 2) (Figure 2).

**Table 2:** Clinical features associated with the cases.

Clinical features	Clinically suspected pyoderma (n = 182)	Bacteriologically confirmed pyoderma (n = 161)
Impetigo contagiosa	62 (34.07%)	56 (34.78%)
Folliculitis	37 (20.33%)	32 (19.88%)
Furunculosis	17 (9.34%)	16 (9.94%)
Cellulitis	2 (1.1%)	2 (1.24%)
Ecthyma	2 (1.1%)	2 (1.24%)
Bullous impetigo	1 (0.55%)	1 (0.62%)
Scabies with secondary infection	27 (14.84%)	23 (14.29%)
Eczema with secondary infection	21 (11.54%)	19 (11.8%)
Infected wound	6 (3.29%)	6 (3.73%)
Insect bite reaction	4 (2.19%)	3 (1.86%)
Contact dermatitis	3 (1.65%)	1 (0.62%)

Among the coagulase-negative staphylococci, the common species isolated were *S. epidermidis* (seven), *S. lugdunensis* (six), *S. haemolyticus *(two), *S. hominis* (two), and *S. saprophyticus* (one). All staphylococci were susceptible to vancomycin, teicoplanin, and linezolid. There were 17 (13.5%) cases of methicillin-resistant *S. aureus* (MRSA). The staphylococci were also most susceptible to fluoroquinolones and doxycycline (Table 3).

**Table 3:** The antibiotic susceptibility pattern of the isolated organisms. NA = not applicable

Antibiotic	*Staphylococcus aureus* (n = 126)	Coagulase-negative staphylococci (n = 18)	*Streptococcus pyogenes *(n = 16)
Penicillin	NA	NA	16 (100%)
Cefoxitin	109 (86.5%)	12 (66.67%)	NA
Erythromycin	78 (61.9%)	12 (66.67%)	10 (62.5%)
Clindamycin	103 (81.75%)	14 (77.78%)	13 (81.25%)
Ciprofloxacin	105 (83.33%)	14 (77.78%)	16 (100%)
Levofloxacin	112 (88.89%)	15 (83.33%)	16 (100%)
Gentamicin	87 (69.05%)	14 (77.78%)	NA
Amikacin	87 (69.05%)	14 (77.78%)	NA
Doxycycline	118 (93.65%)	18 (100%)	NA
Cotrimoxazole	92 (73.02%)	15 (83.33%)	16 (100%)
Linezolid	126 (100%)	18 (100%)	16 (100%)
Vancomycin	126 (100%)	18 (100%)	NA
Teicoplanin	126 (100%)	18 (100%)	NA

There was only one *P. aeruginosa* isolate, which was susceptible to cefepime, piperacillin/tazobactam, cefoperazone/sulbactam, imipenem, meropenem, ciprofloxacin, levofloxacin, and colistin and resistant to ceftazidime, amikacin, and gentamicin.

Of the 126 cultures of *S. aureus* grown from the patients’ pyoderma samples, 73 (57.94%) patients were colonized with it, of whom 64 (50.79%) were colonized with methicillin-sensitive *S. aureus* and nine (7.14%) were colonized with MRSA.

## Discussion

This study, performed in a tertiary care center in Eastern India over a period of one year, included 182 cases of pyoderma in a pediatric population who fulfilled the inclusion criteria. Most (68, 37.36%) of the children were one to four years of age, with 52 (28.57%) in the five-to-eight-year age group. The preponderance of preschool-aged children might be because these children are exposed to unhygienic conditions. Hazarika et al. observed that pyoderma was more frequent in the one-to-five-year age group (52%) [5], and Nagmoti et al. had similar findings, reporting that 45% of the patients with pyoderma belonged to the one-to-four-year age group [6]. Although the number of male patients was slightly higher, there was not much sex predominance. However, Nagmoti et al. observed a higher male preponderance (approximately two-thirds of the included population) [6]. Two-thirds (119, 65.38%) of our patients came from urban areas, a finding similar to that of Hazarika et al. who found that 75% of the population was from urban areas [5]. This could be because the study was performed in a tertiary care center in an urban area.

Most patients presented during the hot and humid months of May (15.52%), June (11.8%), July (13.04%), August (9.93%), and September (10.56%). We also observed a higher prevalence of primary pyoderma in the hot and humid months of May (13.66%), June (11.18%), July (9.93%), August (8.69%), and September (6.83%), whereas secondary pyoderma was more common in the winter months of December (6.84%) and January (6.83%). A study by Banerjee et al. observed that impetigo and furunculosis were more common during the rainy season, whereas scabies was more common during the winter [7].

Primary pyoderma (66.48%) was more common than secondary pyoderma (33.52%). Among the primary pyoderma cases, impetigo contagiosa (34.07%) was the most common, followed by folliculitis (20.33%). Among the secondary pyoderma cases, scabies with secondary infection (14.84%) was more common than eczema with secondary infection (11.54%). In a similar study performed by Singh et al. in Rajasthan, 61% of the cases were primary pyoderma; among these, furuncle (27.2%) was the most common entity, followed by folliculitis (13.2%) and impetigo (9.4%), in contrast to our study, in which impetigo was the most common. Infectious eczematoid dermatitis (12.4%) and infected scabies (6.8%) were the two most common entities among secondary pyoderma [8]. Malhotra et al., however, found that 80.33% of cases were secondary pyoderma [9]. Another study performed in a tribal population found that impetigo contagiosa was the most common presentation [10]. The head and neck (33.52%) were the most common lesion sites, followed by the lower limbs (19.78%). However, Singh et al. found that the lower extremities were the most commonly affected sites [8]. Pustules were the most common type of lesion (61.54%), followed by crusting and erosion (16.48%), and most of the patients presented with the complaints of pain, fever, and discharge (64.83%). Hulmani et al. also found that pustules were the most common presentation, observed in 65% of cases [3].

Pyoderma, impetigo, and impetigo contagiosa are terms used synonymously to describe discrete purulent lesions that are primary skin infections and are extremely prevalent in many parts of the world. In the large majority of cases, pyoderma is caused by β-hemolytic streptococci, *S. aureus*, or both [11]. Of the 182 patients with pediatric pyoderma in our study, 161 samples were bacterial culture-positive and 21 samples were bacterial culture-negative. Of the 161 bacterial culture-positive samples, *S. aureus* growth was observed in 126 (78.26%) samples, followed by coagulase-negative staphylococci (18, 11.18%), *S. pyogenes* (16, 9.93%), and one sample showing *P. aeruginosa *growth (0.62%). Most of the recent studies in India have found that *S. aureus* is the most common cause of pyoderma in children. This finding has also been demonstrated by Hulmani et al. in Davangere (82/92 of positive cultures were *S. aureus*) [3], Ashokan et al. in 55.7% of cases in Nellore [1], and Hazarika in 119/160 cases in Guwahati [5].

Of the 126 *S. aureus* isolates in our study, 17 (13.5%) were MRSA. In a study performed in Kerala by Rani et al., the most common cause of pyoderma was *S. aureus*, with 13% being MRSA [12], which is a strong sign that the prevalence of MRSA was lower. A study by Sengupta et al. observed a high prevalence of MRSA in neonatal omphalitis, which is an alarming sign [13]. *S. aureus* was completely susceptible to vancomycin, teicoplanin, and linezolid, similar to the findings of Sengupta et al. [13]. The MRSA isolates were also highly sensitive to clindamycin, doxycycline, levofloxacin, and ciprofloxacin. Complete knowledge regarding the causative organism, along with its antimicrobial susceptibility pattern, is required to properly manage cases and prevent the spread of antimicrobial resistance.

It has been found that the proportion of MRSA among community-associated *S. aureus* infections in Asian countries varies markedly from <5% to >35% [14]. Of the 126 *S. aureus* cultures grown from the pyoderma samples of our patients, 73 (57.94%) patients were colonized with the bacteria, nine (7.14%) of whom were colonized with MRSA. Nasal colonization with *S. aureus* was observed in 59.7% of the patients in a study performed in Bengaluru [15]. A community-based study performed in India found that the overall prevalence of *S. aureus* nasal colonization was 52.3% and that of MRSA was 3.89% [16].

One limitation of this study was that a molecular characterization of the resistant bacteria was not performed. Another limitation was that most of the patients did not return for follow-up, possibly because they were cured.

## Conclusions

This study showed the pattern of pyoderma in pediatric age groups in a tertiary care center in Eastern India. More patients had primary pyoderma in the form of pustules, with the most common location being the head and neck region. Pyoderma cases were more prevalent in the hot and humid months of May, June, July, and August. The most common cause of pyoderma was *S. aureus*, although the prevalence of MRSA was low, and they were all susceptible to vancomycin, teicoplanin, and linezolid. A thorough understanding of the clinical patterns of pyoderma, its causative agents, and its antimicrobial susceptibility patterns is essential for managing cases and preventing the spread of antimicrobial resistance.
